# Development of Copper-Aluminum Layered Double Hydroxide in Thin Film Nanocomposite Nanofiltration Membrane for Water Purification Process

**DOI:** 10.3389/fchem.2019.00003

**Published:** 2019-02-08

**Authors:** Muhammad Hanis Tajuddin, Norhaniza Yusof, Ihsan Wan Azelee, Wan Norharyati Wan Salleh, Ahmad Fauzi Ismail, Juhana Jaafar, Farhana Aziz, Kazukiyo Nagai, Nor Faizah Razali

**Affiliations:** ^1^Advanced Membrane Technology Research Centre (AMTEC), Universiti Teknologi Malaysia, Skudai, Malaysia; ^2^School of Chemical and Energy Engineering, Faculty of Engineering, Universiti Teknologi Malaysia, Skudai, Malaysia; ^3^Department of Applied Chemistry, Meiji University, Kawasaki, Japan; ^4^Faculty of Engineering Technology, Universiti Tun Hussein Onn Malaysia, Parit Raja, Malaysia

**Keywords:** Cu-Al layered double hydroxides, polyamide, thin film composite membrane, nanofiltration, anti-fouling

## Abstract

This study aims to fabricate a thin film composite (TFC) membrane, modified with copper-aluminium layered double hydroxide (LDH) nanofillers via interfacial polymerization technique for nanofiltration (NF) processes. It was found that Cu-Al LDH nanofillers possessed layered structured materials with typical hexagonal plate-like shape and positive surface charge. The study revealed that TFN membrane exhibits a relatively smooth surface and a less nodular structure compared to pristine TFC membrane. The contact angle of TFN progressively decreased from 54.1° to 37.25°, indicating enhancement in surface hydrophilicity. Moreover, the incorporation of LDH nanofillers resulted in a less negative membrane as compared to the pristine TFC membrane. The best NF performance was achieved by TFN2 membrane with 0.1° of Cu-Al LDH loading and a water flux of 7.01 Lm-2h-1.bar. The addition of Cu-Al LDH resulted in excellent single salt rejections of Na_2_SO_4_ (96.8%), MgCl_2_ (95.6%), MgSO_4_ (95.4%), and NaCl (60.8%). The improvement in anti-fouling properties of resultant TFN membranes can be observed from the increments of pure water flux recovery and normalized water flux by 14% and 25% respectively. The findings indicated that Cu-Al LDH is a promising material in tailoring membrane surface properties and fouling resistance. The modification of the LDH-filled TFN membrane shows another alternative to fabricating a high-performance composite membrane, especially for water softening and partial desalination process.

## Introduction

Global water scarcity has affected people in the twenty-first century as the present water resource is insufficient to provide enough clean water, especially in the urban and water-stressed region. Therefore, with the latter problem address previously, technologies such as membrane separation process has serve as one of the alternatives to overcome this issue. In view of this aforementioned problem, nanofiltration (NF) which is a type of membrane separation process where its performance and characteristic pore lie between ultrafiltration and reverse osmosis has been employed for this purpose. Generally, NF membranes possess a pore size of 1 nm and a low molecular weight cut-off of 100–1,000 Da that is suitable for effective divalent salts separation (Li et al., 2014; Ma et al., 2017). The fabrication of thin film composite (TFC) membrane by interfacial polymerization has provided significant advantages owing to its unique structures such as ultra-thin selective layer and porous substrate of TFC membrane. Both layers [polyamide (PA) thin layer and substrate layer] can be independently optimized and controlled to achieve desired selectivity while retaining the excellent mechanical strength and compression resistance. These intrinsic properties allow the fabricated TFC membrane to achieve high water flux and salt rejection.

However, one of the major drawbacks of TFC membranes is their performance is largely controlled by their water permeability, solutes selectivity, and fouling resistances. Due to these circumstances, the TFC membrane suffers from selectivity/permeability or trade-off effects in achieving the excellent performance of a NF separation process. Many attempts have been made to create a TFC membrane with high water permeation without sacrificing the salts retention (Lau et al., 2015; Wang et al., 2017; Zhu et al., 2018). In the past years, various approaches have been employed to improve water flux and separation performance of TFC through the use of novel monomer (Ismail et al., 2015) or through incorporating inorganic nanofiller (Yin and Deng, 2015) either in PA layer or substrate layer. Due to that, thin film nanocomposite membranes (TFN) have been proposed as a new way to develop high performances of composite membrane. With the addition of nanofillers into the membrane, membrane properties can be improved which results in higher membrane hydrophilicity or additional pathways for water transportation across membrane structures.

Layered double hydroxide (LDH) is a group of two-dimensional anionic clays made up from positively charged metal ions and an interlayer gallery composed of anions and water molecules. The general formula of LDH structures is [M(II)_1−x_M(III)_x_(OH)_2_]^x+^[(A^n−^)_x/n_.yH_2_O], where M(II) is divalent cation Mg^2+^, Zn^2+^, Ca^2+^, Cu^2+^ etc., M(III) is trivalent cation Al^3+^, Fe^3+^ and Cr^3+^, and A^n−^ is interlayer anion (Tajuddin et al., 2018). Most of LDH configurations have an octahedral structure where metal cations are at the centers of the edge sharing octahedral with each metal contain six OH- ions (Mishra et al., 2018). Besides that, the divalent and trivalent ions are distributed uniformly in the hydroxide layer structures. With special properties such as high anion exchanges, hydrophilic in nature (Ghani et al., 2018), and made up of a combination of metals from copper and aluminum materials, they possess high thermal conductivity (Sarkar et al., 2017) which is beneficial for an extreme conditions separation. One of the major drawbacks of neat TFC membrane is its highly negative charged surface, which will lead to fast deposition of cationic foulants (Dong et al., 2015). With the incorporation of Cu-Al LDH a positive charged filler is beneficial for the separation of cationic foulants when embedded in the membrane matrix.

Thus, the purpose of this present work is to develop a composite membrane for high performances in water permeability improves against inorganic salts rejection and anti-fouling by embedding Cu-Al LDH nanofillers into PA layer. The incorporation of Cu-Al LDH nanofillers in the membrane structure was analyzed in terms of surface morphology, hydrophilicity, zeta potential, and chemical functional group. The separation performance of neat TFC and TFN membranes were systematically investigated through single rejections against inorganic salts and cetrimonium bromide (CTAB) rejections.

## Materials and Methods

### Materials

Commercial US020 polysulfone (PSf) membranes with a molecular weight cut-off of 20,000 g/mol were provided by Rising Sun Membrane to be used as the substrate. Piperazine (PIP), trimesoyl chloride (TMC), and n-hexane (purity >99.5%) were obtained from Merck and were used, respectively, as the aqueous monomer, organic monomer, and organic solvent. Copper (II) nitrate trihydrate Cu(NO_3_)_2_.3H_2_O, aluminum nitrate nonahydrate Al(NO_3_)_3_.9H_2_O powder, and sodium hydroxide (NaOH) were purchased from Sigma Aldrich and Merck, respectively. All chemicals were of analytical grade and used without further purification. For membrane performance evaluation, Na_2_SO_4_, MgSO_4_, MgCl_2_, and NaCl supplied by Sigma Aldrich were used as the single inorganic salt solution. For fouling study, CTAB was used as a cationic model foulant and was supplied by Sigma Aldrich.

### Cu-Al LDH Synthesis

Cu-Al LDH was synthesized by using co-precipitation method, according to the procedure of Meng et al. (2017). Briefly, a 0.1 mol/L Cu(NO_3_)_2_ solution was mixed with 100 ml of 0.2 mol/L NaOH to precipitate Cu(OH)_2_. Then, the newly prepared Cu(OH)_2_ precipitate was added to a 100 mL of 0.05 mol/L Al(NO_3_)_3_ solution, under 60 min of vigorous stirring. The pH was adjusted to 12 by adding a 1 mol/L NaOH solution to allow the reaction between the two hydroxides. The suspension obtained was aged for 6 h and then separated through centrifugation at 7,000 rpm before being washed with deionized water several times to purify the LDH until the pH was 7. After that, the samples were further washed with acetone to minimize agglomeration (Lu et al., 2016). Finally, the obtained LDH powder was dried at 60°C for 24 h and stored until further use.

### Cu-Al LDH Nanofillers Characterizations

Transmission electron microscopy (TEM) (JEOL JEM-ARM 200F) was employed to observe the structure of Cu-Al LDH fillers and was operated with an accelerating voltage of 200 kV. Prior to analysis, an appropriate amount of nanofillers was ultrasonicated in an alcohol solution and a drop of solution was deposited on a copper mesh grid. The zeta potentials of the Cu-Al LDH were diagnosed by using a Zetasizer 3000HSA (Malvern Instruments) with water as a dispersant at pH = 7.

### Preparation of Thin Film Nanocomposite Membrane

The TFC and TFN membranes were polymerized on top of PSf substrate. The PSf substrate was soaked in deionized water 24 h prior to use. The PSf commercial substrate membrane was clamped firmly between a glass plate and a viton rubber sheet with a square window. The composition of the organic phase solution was 0.1 (w/v)% TMC while the aqueous solution was kept at 2 (w/v)% PIP. The aqueous solution was poured on top of the PSf substrate and was allowed to soak for 2 min. The excess PIP solution was drained off gently from the surface by using a rubber roller and tissue. Then, the organic solution containing TMC was contacted with the amine saturated substrate and was kept for 1 min, which *in-situ* interfacial polymerization take place simultaneously and cured in an oven at 60°C for 5 min, followed by rinsing and storage in DI water prior to usage. Similar steps were applied to fabricate the TFN membrane by adding a certain amount of Cu-Al LDH powder in the organic solution where the composition of the amine solution and reaction conditions were equal to that of a pristine TFC membrane. The resulting membranes were designated as TFC, TFN 1, TFN 2, TFN 3, and TFN 4, respectively, according to the composition given in Table 1.

**Table 1:** Composition of the amine solution in aqueous and acid chloride in organic phase and LDH loading.

**Membrane**	**PIP (w/v %)**	**TMC (w/v %)**	**LDH loadings in TMC solutions (wt %)**
TFC	2.0	0.1	0
TFN 1	2.0	0.1	0.05
TFN 2	2.0	0.1	0.1
TFN 3	2.0	0.1	0.15
TFN 4	2.0	0.1	0.2

### Thin Film Composite/Nanocomposite Membranes Characterizations

The chemical structures of the TFC and TFN membranes were analyzed by using an ATR-FTIR spectrometer (Model: IRTRACE100, Shimadzu). The membrane sample (1 mg) was cut into small pieces and loaded into IR disks and subsequently scanned by a single-reflectance ATR accessory with IRTRACER100. Each sample was scanned in a range of 4,000–500 cm^−1^ with a scanning resolution of 0.25 cm^−1^. A field-emission scanning electron microscopy (FE-SEM) (Model: Hitachi SU8020) was used to identify the morphologies of the TFN membranes. Different FESEM micrographs with various magnifications (10.0 and 50.0 k) of the surface of the TFC/TFN membranes were obtained. The zeta potential analysis of the TFN membranes was carried out by using an electrokinetic analyzer (Model: Malvern Zetasizer Nano Zsp) equipped with an adjustable gap cell based on streaming potential and streaming current measurement. Prior to measurement, the membrane samples were immersed in RO water for at least 12 h to complete hydration. Two membrane samples of 20 × 10 mm were placed on the sample holder using a double-sided tape. The membrane hydrophilicity was measured by contact angle using a static goniometer (Kruss Gambult, Germany). A 1.00 μL of water droplet was placed onto the top surface of the membrane sample from the motor-driven syringe and the contact angle was measured. Reading was repeated at least 15 times on different locations of the membrane and the average value was reported.

### Nanofiltration Performances

The nanofiltration performance of fabricated TFC and TFN membranes was evaluated in terms of water permeability and single salt rejection by using a lab scale cross-flow filtration system (Model: Sterlitech™ HP4750 Stirred Cell) with an effective surface area of membrane of 20.6 cm^2^. Prior to flux measurement, all membrane samples were compacted for 30 min at 8 bar to reach steady state. Then, the operating condition was maintained at 7 bar and 25°C. The flux and salt rejection were determined using 1,000 ppm of different inorganic salts, including Na_2_SO_4_, MgSO_4_, MgCl_2_, and NaCl.

$$\begin{array}{l} J=\frac{V}{t\times A} \end{array}$$

where *V* is permeate volume (L), *A* is membrane area (m^2^), and *t* (t) is time required to collect the permeate volume *V*. The salt concentration in the feed and permeate solutions were measured by using a bench conductivity meter (Model: Jenway 4520). The salt rejection was calculated by using the equation:

$$\begin{array}{l} R\left(\%\right)=\left(1-\frac{C_{p}}{C_{f}}\right)\times 100 \end{array}$$

where *C*_*p*_ is permeate salt concentration (ppm) and *C*_*f*_ is feed salt concentration (ppm), respectively.

Antifouling experiments were conducted by using 500 ppm CTAB as the model cationic foulants solution. The fouling experiments were carried out at operating pressure of 7 bar during the whole process. The normalized flux (*J*_*f*_*/J*_0_) was used to analyze the fouling behavior of the tested membrane, where *J*_*t*_ and *J*_0_ are fluxes at operational period *t* and *0*, respectively. Then, each membrane was washed by immersing the membrane in a beaker of deionized water and shaken for 15 min to remove the residual of CTAB on the membrane surface before the water flux was measured again.

## Results and Discussion

### LDH Morphological Structures

**Figure 1:**
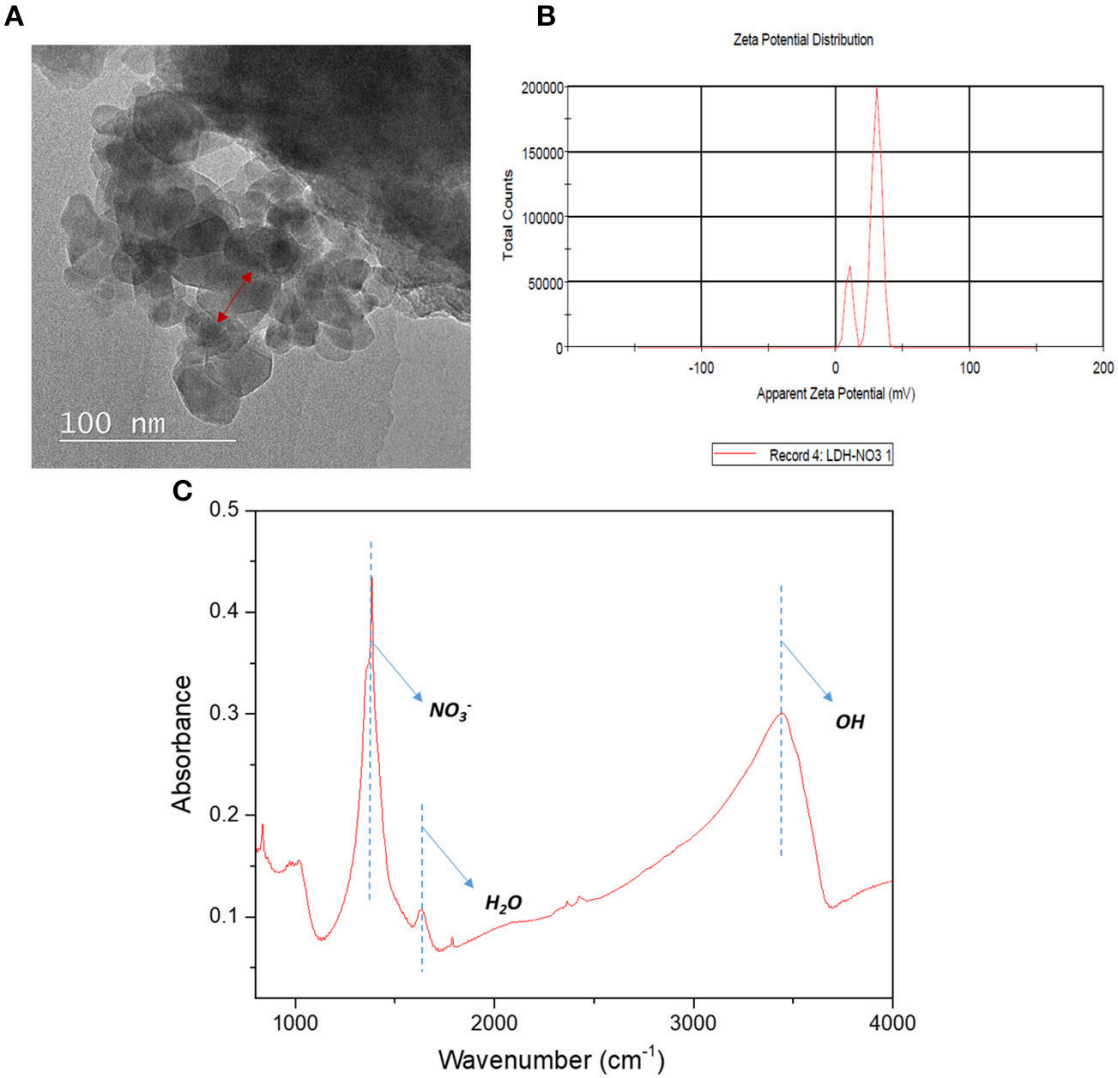
**(A)** Morphological structures **(B)** Zeta potential analysis of Cu-Al LDH **(C)** FTIR analysis of Cu-Al LDH in 4,000–500 cm^−1^ region.

Figure 1 depicts the morphological structures of Cu-Al LDH nanofillers from the TEM analysis. The structures of LDH nanofillers appear to be aggregated and stacked together due to their high surface charge and surface tension (Lu et al., 2017). The TEM image obtained revealed the irregular hexagonal morphology was more distinguishable. As can be seen from Figure 1B, the surface zeta potential of LDH exhibits a positive value of 26.5 ± 3.7 mV. The positive zeta potential value of Cu-Al LDH are largely controlled by the presence of aqueous solution that contains electrolyte. Due to the previously mentioned condition, LDH exhibited the formation of an electrical double layer on its surface. The formation of an electrical double layer in the LDH can be distinguished by two layers (Chen et al., 2017). The first layer exists due to ions adsorbed through different chemical surface interactions such as electrostatic forces and hydrogen bonds while the second layer forms due to the influence of electrical attraction and thermal motion from weak electrostatic forces of free ions present in the fluid (Gondim et al., 2018). The chemical functional groups of Cu-Al LDH fillers in Figure 1C were evaluated by using FTIR analysis. The strong characteristics bands at 3,447 cm^−1^ of Cu-Al LDH can be assigned to the stretching mode of OH groups in brucite layers meanwhile the peak at 1,387 cm^−1^ belongs to the ${\text{NO}}_{3}^{2-}$ ions in the layer structure and the peak at 1,645 cm^−1^ corresponds to the interlayer water molecules in LDH (Peng et al., 2018).

### Characterizations of TFC/TFN Membranes

**Figure 2:**
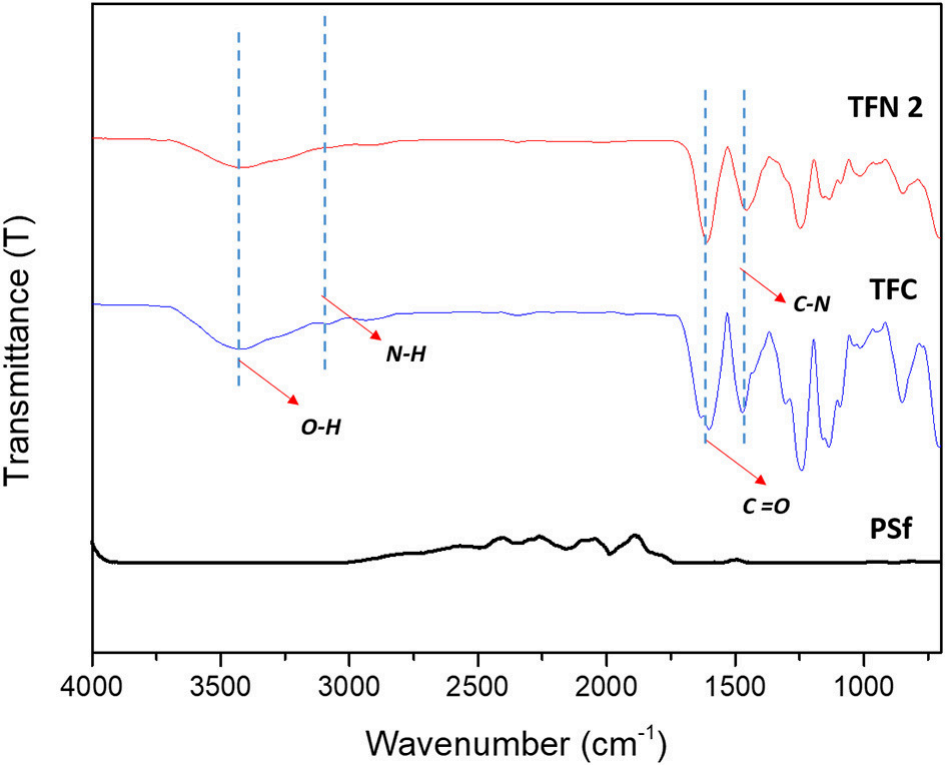
Chemical functional group by FTIR of PSf, TFC, and TFN membranes.

**Figure 3:**
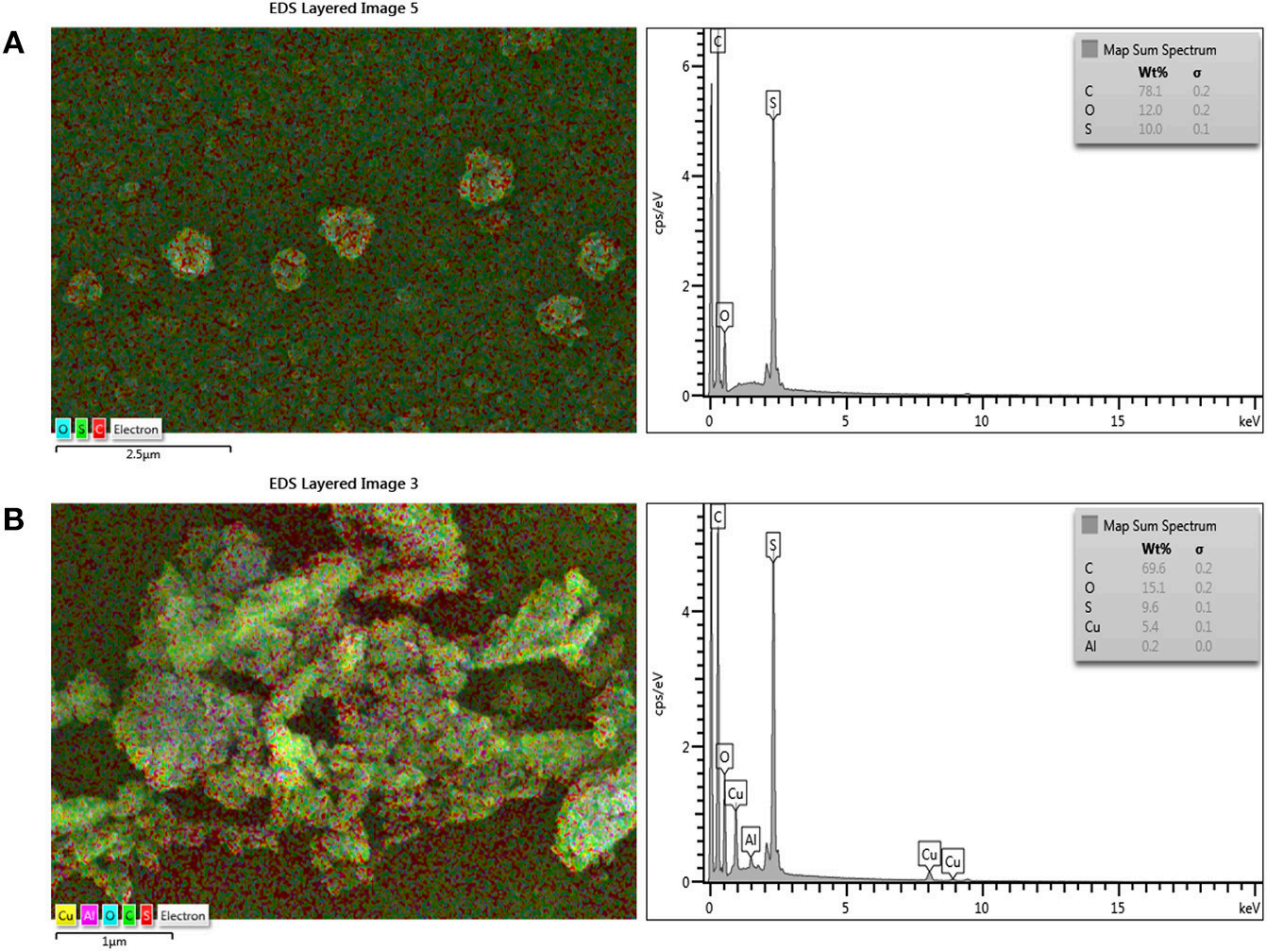
EDX analysis of **(A)** TFC and **(B)** TFN 2 membranes.

Figure 2 shows the chemical surface structures of pure PSf substrate, and TFC and TFN membranes that were analyzed using FTIR spectra. The characteristic bands for a mixture of C-N stretching and NH deformation plane amide (III) was detected at a wavenumber of 1,429 cm^−1^, while 1,610 cm^−1^ indicates the presence of stretching vibration of C = O amide group. The broad adsorption band at 3,250 cm^−1^ can be assigned to N-H stretching vibration and the characteristic peak at 3,419 cm^−1^ is attributed to OH functional group. In comparison to PSf substrates, these bands were not distinguished. These features are similar to the thin film membrane fabricated by Li et al. (2016, 2017) and Yahia Cherif et al. (2018). All these characteristic bands show that the PA layer has been successfully formed on the surface of the PSf substrate. However, the FTIR spectrum of TFC and TFN membranes display almost similar pattern, probably due to the relatively lower loading of Cu-Al LDH. The incorporation of Cu-Al LDH in composite membrane can be further supported with EDX mapping of both TFC and TFN membranes in Figure 3. From Figure 3B, it can be seen that the TFN membrane shows the presence of copper or aluminum in their membrane structures meanwhile the TFC membrane does not possess any trace of those metal ions.

**Figure 4:**
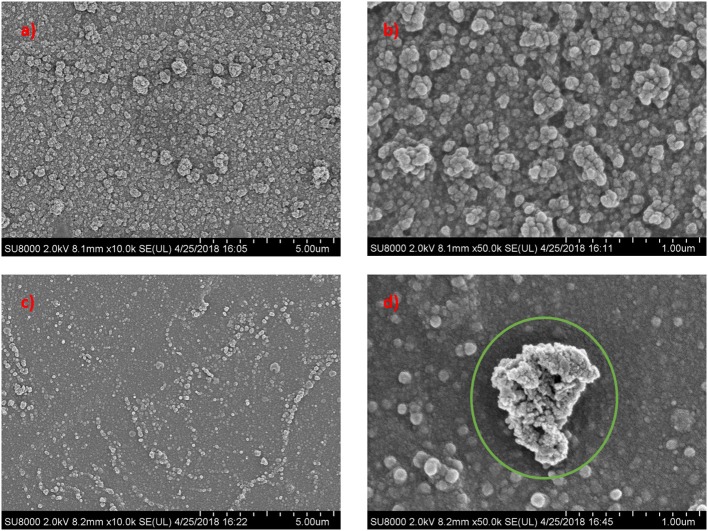
Surface morphological structures of pristine TFC and LDH-filled TFN membrane at different magnifications **(a)** TFC **(c)** TFN 2 at 10 k magnification; **(b)** TFC **(d)** TFN 2 at 50 k magnification.

Figure 4 displays the FESEM images of top surface structures for neat TFC and TFN membranes. The FESEM analysis was done for two membranes for comparison between TFC and TFN membranes. It was found that TFC and TFN 2 revealed the typical nodular structure and spherical globules formed via the interfacial polymerization process between PIP and TMC (Zhu et al., 2018). However, the surface of the TFN membrane became relatively smoother in comparison with pristine TFC, which is mainly attributed to decreased formation rate of nodules and spherical globules on the PSf substrate after the introduction of Cu-Al LDH in the active layer. Moreover, this observation is most probably related to the swelling property of the layered structure in its configuration (Zhao et al., 2016).

**Figure 5:**
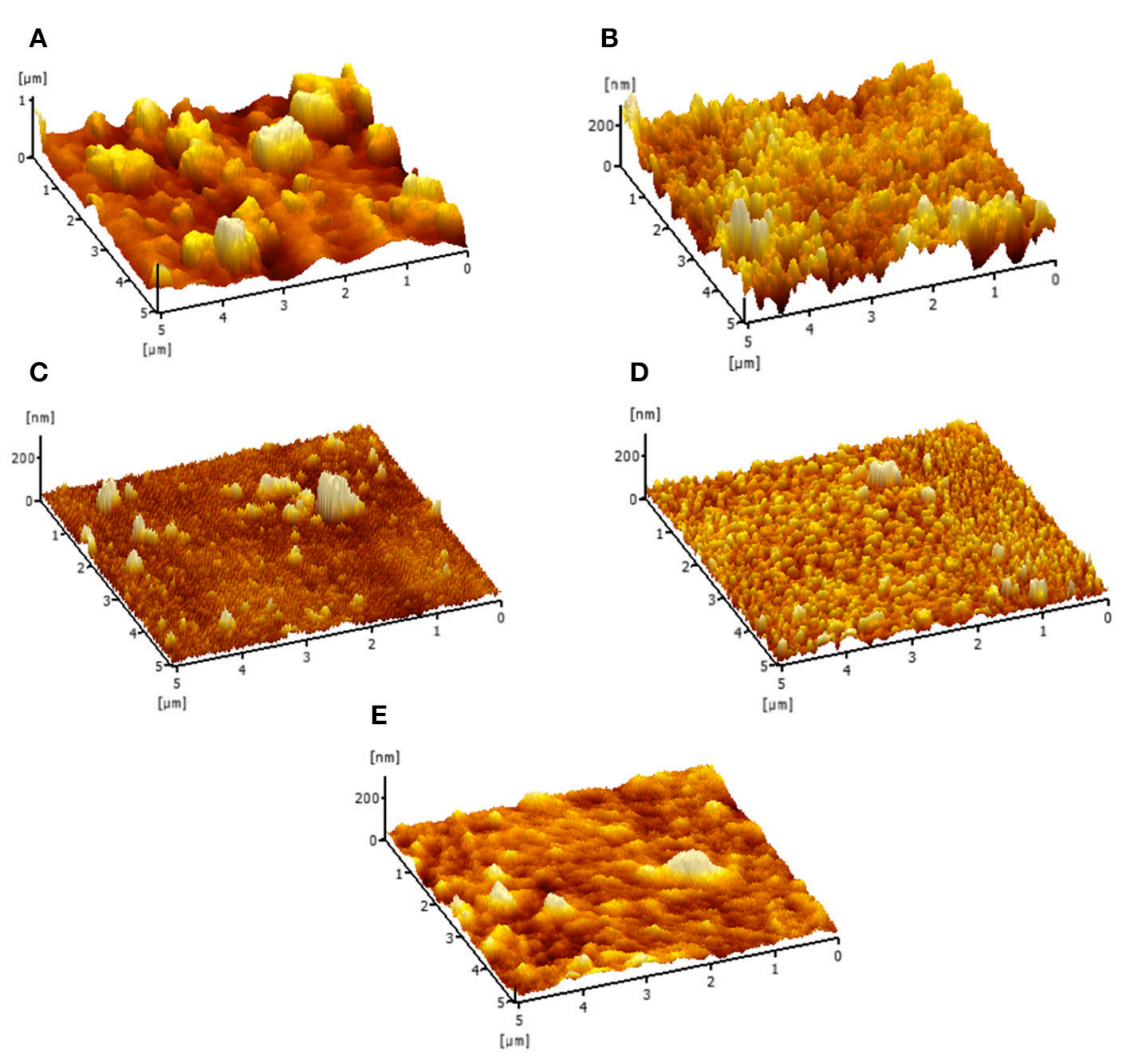
Membrane surface roughness of **(A)** TFC **(B)** TFN 1 **(C)** TFN 2 **(D)** TFN 3 and **(E)** TFN 4 membrane.

The AFM analysis was conducted to evaluate the surface roughness of these membranes and the results were tabulated in Figure 5 and Table 2. From the Table 2, the TFC membrane has a R_a_ (average roughness) of 80.16 nm. However, after the incorporation of Cu-Al LDH in the polyamide layer, the membrane surface becomes relatively smoother. The average roughness of TFC and TFN membranes can be deduced in this order TFC (80.16 nm) > TFN 1 (31.88 nm) > TFN 2 (20.53 nm) > TFN 3 (18.46 nm), and TFN 4 (11.16 nm). As for TFC, these phenomena closely related with the diffusion rate during the interfacial polymerization process of PIP and TMC. PIP monomers have a smaller molecular weight in which during these process, it can easily permeate through the interface of aqueous and organic phase thus resulting in rougher surface roughness (Sun and Wu, 2018). Meanwhile in case of TFN membranes, the differences in surface properties could be attributed due to the lower diffusion rate caused by Cu-Al LDH during the interfacial polymerization which leads to smoother surface roughness. Membranes with a low surface roughness are more preferred in water purification applications since it have strong anti-fouling properties, mainly came from its hydrophilic in nature and smoother surface.

**Table 2:** Surface zeta potential of pristine TFC and TFN membranes.

**Membranes**	**Surface zeta potential (mV)**
TFC	−17.0
TFN 1	−16.5
TFN 2	−11.8
TFN 3	−13.7
TFN 4	−10.2

**Figure 6:**
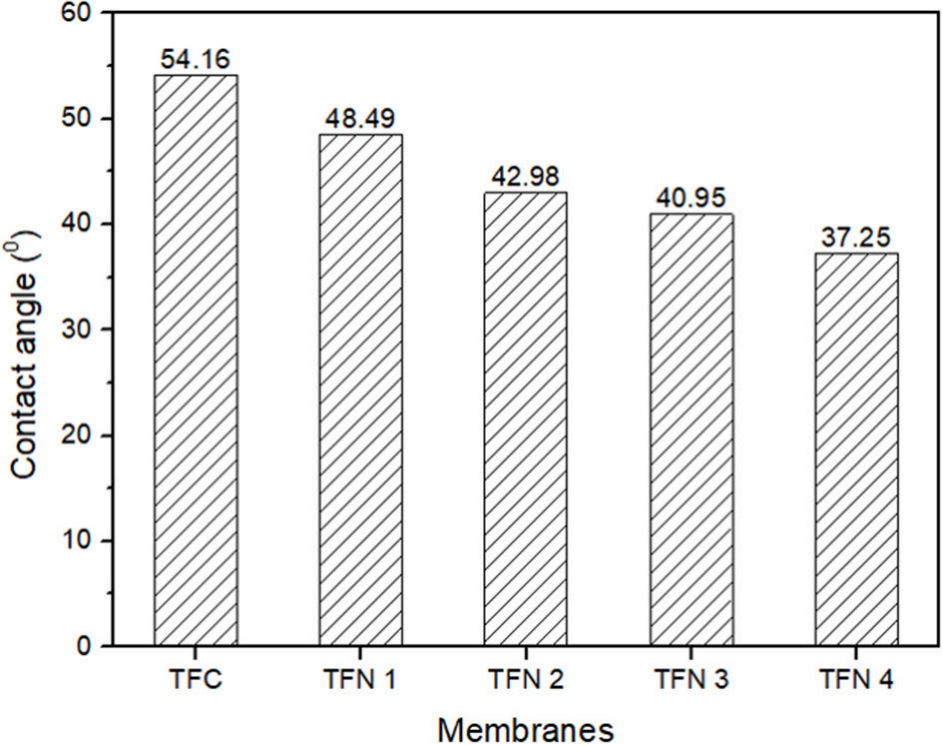
Membrane surface hydrophilicity.

The membrane hydrophilicity of TFC and TFN membranes is presented in Figure 6. As shown, the contact angle has progressively decreases from 54.6 of the TFC membrane to 37.2, as the loading of LDH increases. The improvement of membrane hydrophilicity is mainly attributed to the hydrophilic property and hydroxyl functional groups in LDH as they might play a significant role in the enhancement of water permeability of membranes. As the membrane become more hydrophilic, it can attract more water molecules on the membrane surface thus resulting in formation of water layer in which can inhibit the attachment of foulants. Besides that, better fouling resistance could also be achieved as hydrophilic membranes reduce the attachment of potential hydrophobic foulants probably caused from the formation of the water layer on the surface of membranes (Gu et al., 2018). As the membrane become more hydrophilic, it can attract more water molecules on the membrane surface thus resulting in formation of water layer in which consequently inhibit the attachment of foulants.

The surface profiles of TFC and TFN membranes are presented in Table 3. All membranes exhibited negative surface charge (−17.0, −16.5, −11.8, −13.7, and −10.2 mV). It is noticeable that the pristine TFC membrane has a more negative zeta potential value than all TFN membranes and this mostly contributed to the presence of carboxylic acid in the PA layer structure (Lu et al., 2017). However, the decrease in zeta potential value after the addition of LDH possible of protonation of hydroxyls group (–${\text{OH}}_{2}^{+}$) in the LDH configurations (Tran et al., 2018). Besides that, from the FESEM image in section Characterizations of TFC/TFN Membranes there is possibility that some of the nanofillers is partially exposed on the outer surface of the membrane that may also play role in providing additional positive-charge on the TFN membrane (Dong et al., 2015; Zhao et al., 2016). Generally, a PA membrane is susceptible toward cationic fouling due to negatively charged surface properties, hence less negative surface charged membrane can prevent the adhesion of foulants on their surfaces, consequently improve the anti-fouling properties of membranes.

**Table 3:** Membrane surface roughness TFC and TFN membranes.

**Membranes**	**R_**a**_ (nm)**
TFC	80.16
TFN 1	31.88
TFN 2	20.53
TFN 3	18.46
TFN 4	11.16

### NF Separation Performances

#### Water Flux and Salts Rejection

**Figure 7:**
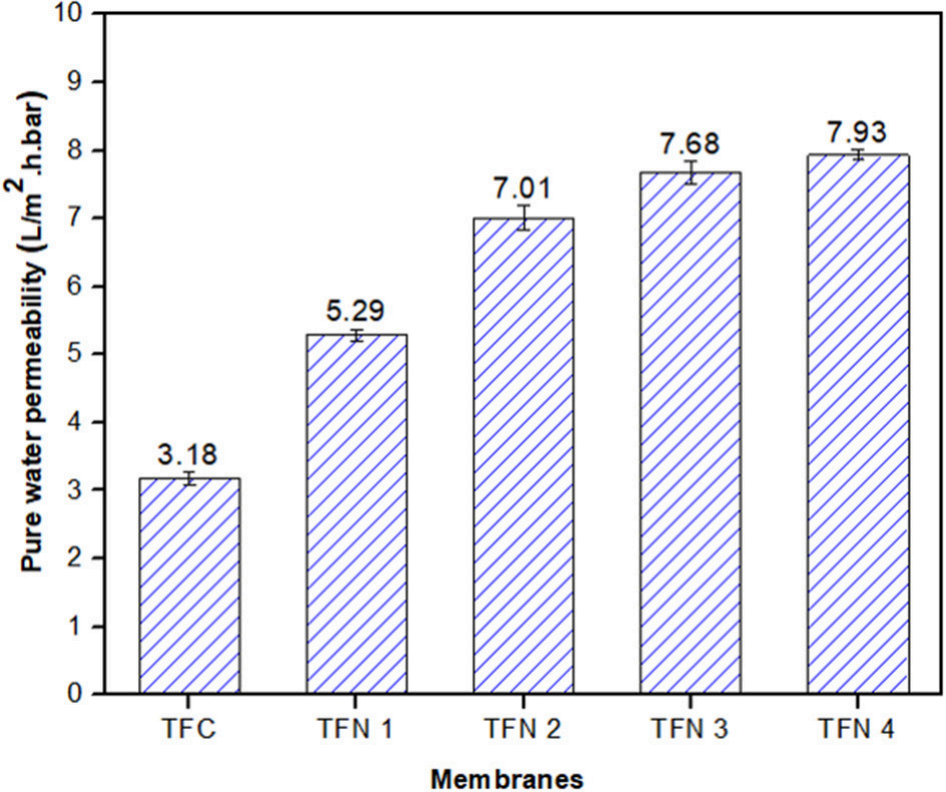
Water permeability of TFC and TFN membranes.

The pure water permeability of composite membranes is displayed in Figure 7, with various loadings. As shown in the figure, the pure water permeability (PWP) of membrane increases from 3.18 to 7.93 L/m^2^.h.bar with increasing Cu-Al LDH loadings from zero to 0.20 wt%, showing a remarkable increment by 149%. The enhancement of PWP could be attributed to improved membrane hydrophilicity upon the addition of LDH into the polyamide layer structure. Moreover, as the LDH possesses unique structures such as an interlayer gallery which has abundant hydroxyls groups, not only can it provide additional room for water molecules but it can also facilitate and add more adhesion to the surface of the membrane (Tian et al., 2018).

**Figure 8:**
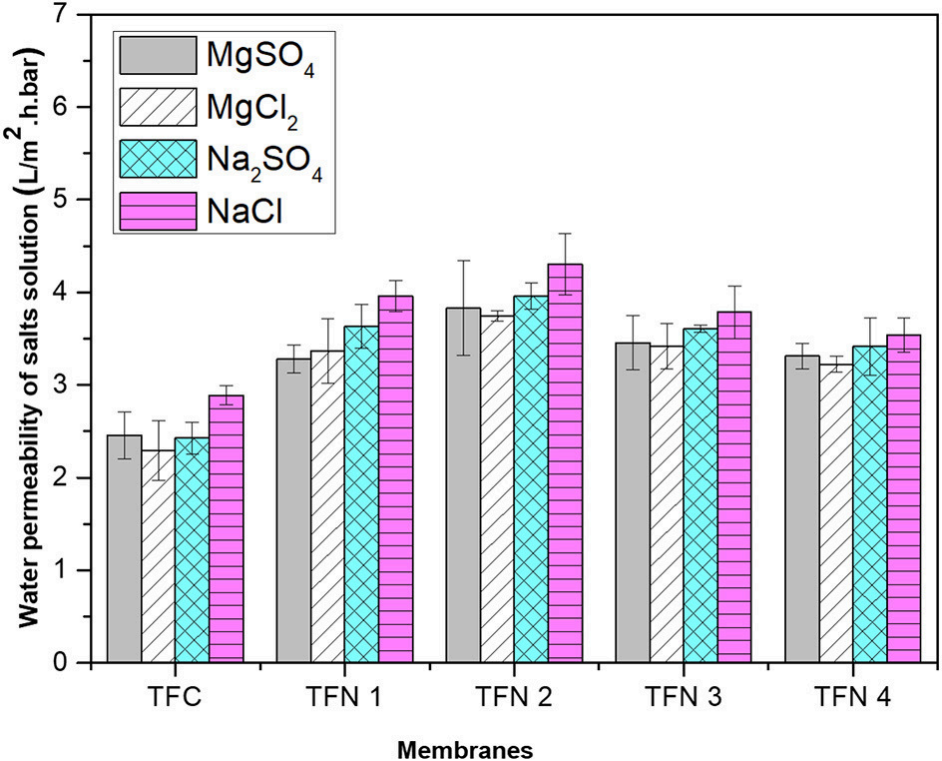
Water permeability of salts solution of different aqueous salt solutions (MgSO_4_, MgCl_2_, Na_2_SO_4_, and NaCl).

The water permeability of aqueous salt (MgSO_4_, MgCl_2_, Na_2_SO_4_, and NaCl) solutions are presented in Figure 8. From the graph, it can be observed that the water permeability of salt solutions of TFC and TFN membranes increased as the loading of LDH was increased from 0.05 to 0.2. Besides that, it can be seen that the permeation flux of solutes is lower than the pure water flux, which is probably attributed to concentration polarization that is mainly caused by salt rejection (Zhu et al., 2018). The water permeability of salt solutions decreased with LDH high than 0.2 most probably due to the external polarization caused by precipitation salts in the PA layer. This precipitation will eventually block the water pathway that was created by the nanofiller itself. As the loadings of nanofillers are increased, it will create more pathway which leads to salts precipitation in the PA layer. The presented data are consistent with the salt rejection that will be discussed in the next section, whereas permeability of solutes increases, rejection slightly decreases for higher loading membranes. Meanwhile, the solute permeability for TFC membranes for all salts is lower compared to the TFN membranes.

**Figure 9:**
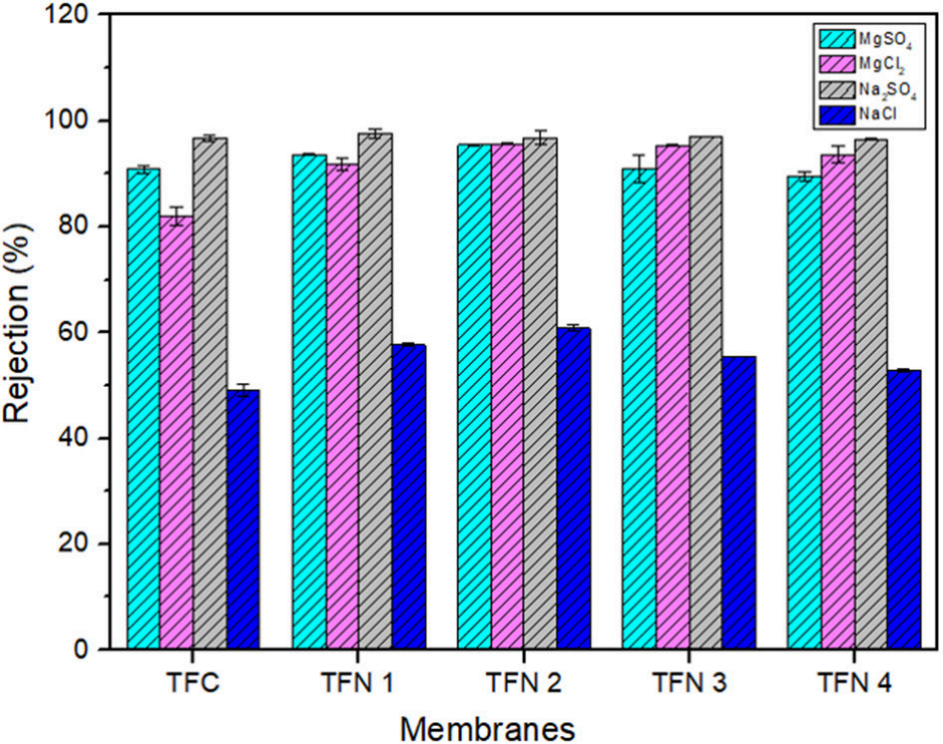
Single salt rejection of TFC and TFN membranes.

Figure 9 represents the full range rejection of different inorganic salts (MgSO_4_, MgCl_2_, Na_2_SO_4_, and NaCl) by TFC and TFN membranes. From Figure 9, these fabricated NF membranes maintained high rejection against divalent salts (Na_2_SO_4_ > MgCl_2_ ≈ MgSO_4_). Their rejections against NaCl are low (45–60%) compared to other inorganic salts. The order of salt rejection demonstrated in this experimental study can be explained by the higher rejection against divalent salts (e.g., MgSO_4_, MgCl_2_, and Na_2_SO_4_) in comparison with monovalent salt (NaCl), which is the typical rejection characteristics of negatively charged NF membranes (Akbari et al., 2016; Lai et al., 2018). Since the fabricated TFN membranes possess less negative surface charged, therefore, the Donnan effect was not dominant in separation of inorganic salts meanwhile sieving effects (Huang et al., 2018) and diffusivity could play another role in rejecting the salts (Rezania et al., 2018). This could be explained by observing the separation behavior of sodium sulfate salts which had a higher rejection than chloride salts. Besides that, the diffusion coefficients of ions vary in order of Cl^−^ (2.032 × 10^−3^ mm^2^ s^−1^) > Na^+^ (1.333 × 10^−3^ mm^2^ s^−1^) > ${\text{SO}}_{4}^{2-}$ (1.065 × 10^−3^ mm^2^ s^−1^) > Mg^2+^ (0.706 × 10^−3^ mm^2^ s^−1^) (Wang et al., 2005; Xie et al., 2017) which further caused the lower rejection of NaCl compared with other salts. In addition, monovalent salts has higher diffusivity compared with divalent salts as a results it can easily penetrate into the membrane which consequently affected the salts rejection. Based on Figure 9, it was found that the salt rejection of the fabricated membranes had slightly increase from the TFC to the TFN2 membrane. These increments could probably be attributed to the properties of LDH, where it can attract more ions into its interlayer region thus significantly improve the rejection of resultant membrane (Lu et al., 2018). However, at higher loadings, the salt rejection slightly decreases, probably due to the excess LDH in polyamide providing an additional barrier and formation defects in PA layer, which subsequently causes the rejection to drop. From the experimental data, it can be concluded that TFN 2 exhibits the best performance, considering its enhancement in water permeability by 120% and their salts rejection.

#### Anti-fouling Study

**Figure 10:**
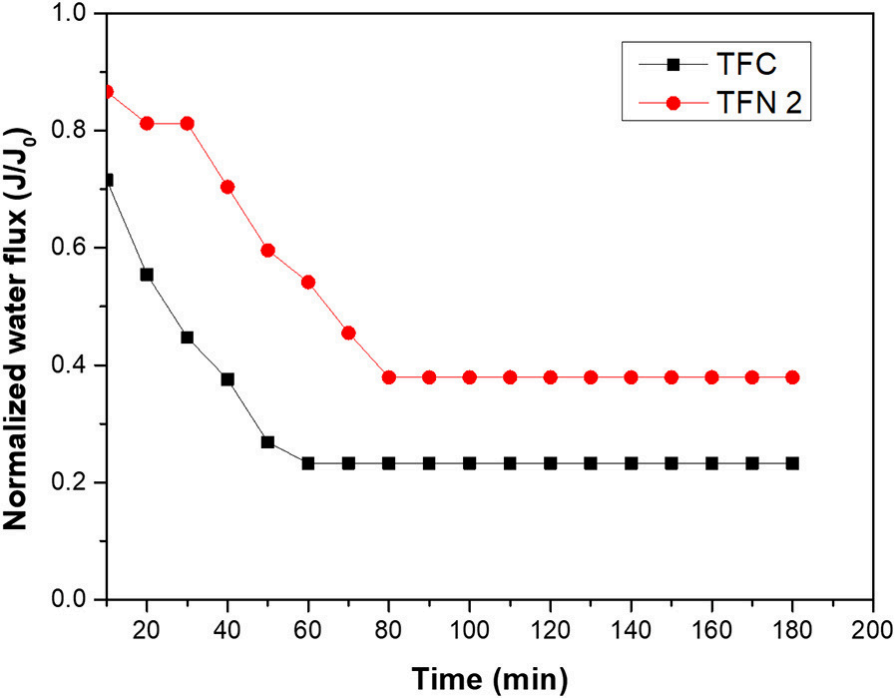
CTAB filtration of pristine TFC and TFN 2 membranes.

To further understand the fouling study of the LDH-filled membrane and the pristine membrane, fouling was evaluated by using CTAB as a cationic surfactant and a graph of CTAB filtration over time is presented in Figure 10. The fouling behavior was studied in the presence of a 500 ppm CTAB solution. For both membranes, fouling instantly took place within a few minutes with an instant drop of normalize water flux. After 3 h of CTAB filtration, the normalized flux of TFC and TFN 2 reduced to 25 and 38%, respectively, which shows that TFN 2 was less fouled. The severe fouling occurred might probably due to strong electrostatic interaction between the membrane and cationic surfactant, resulting in fast deposition on the surface of the membranes which significantly reduced the water flux. The water flux recovery after washing proves that the LDH-filled membranes had less coverage of cationic foulant on their membrane surfaces. The enhancement in fouling study of this membrane can be linked to the zeta potential analysis, which shows that the addition of LDH in TFN membranes rendered the membrane to become less negative. Thus, it is likely that the electrostatic interaction between foulants and membrane surfaces was reduced and less CTAB molecules were attached on the surface of TFN membrane as compared to control.

**Figure 11:**
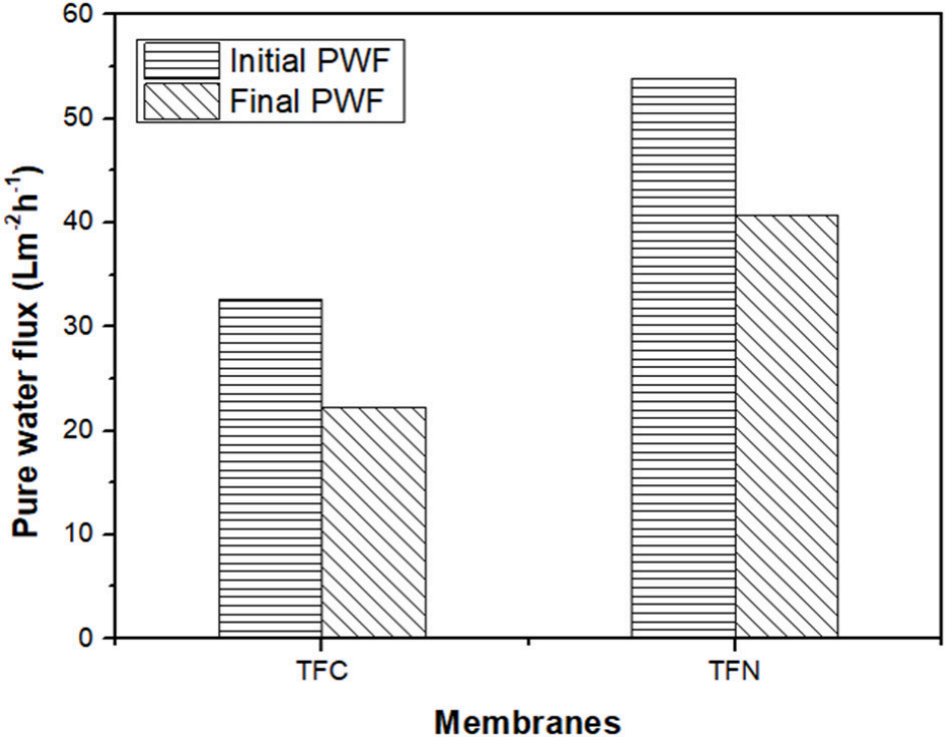
The pure water flux for TFC and TFN 2 before and after CTAB filtration.

The fouling of TFC and TFN was further investigated by measuring pure water flux recovery after the CTAB filtration. Figure 11 displays the pure water flux of pristine and LDH-filled TFN membranes after the filtration of the CTAB solution. As can be seen from the Figure 11, the pristine TFC membrane shows a significant 69% reduction of water flux. The LDH-filled TFN membrane presents a lower drop of water flux of 54% after CTAB filtration. The differences in pure water flux omitted by TFC and TFN membranes are mainly attributed to the presence of LDH nanofillers in selective layers to better mitigate the fouling and improve membrane hydrophilicity. As mentioned previously from the FTIR results, there was a presence of O-H band for TFN 2 that resulted in the high hydrophilicity of the membrane surface. The attachment of foulants is somehow reduced due to the presence of OH groups that attract water molecules instead of foulant compounds. However, the decrease in water flux for both TFC and TFN membranes is probably due to the attachment of irreversible foulants in the membrane selective layers.

**Figure 12:**
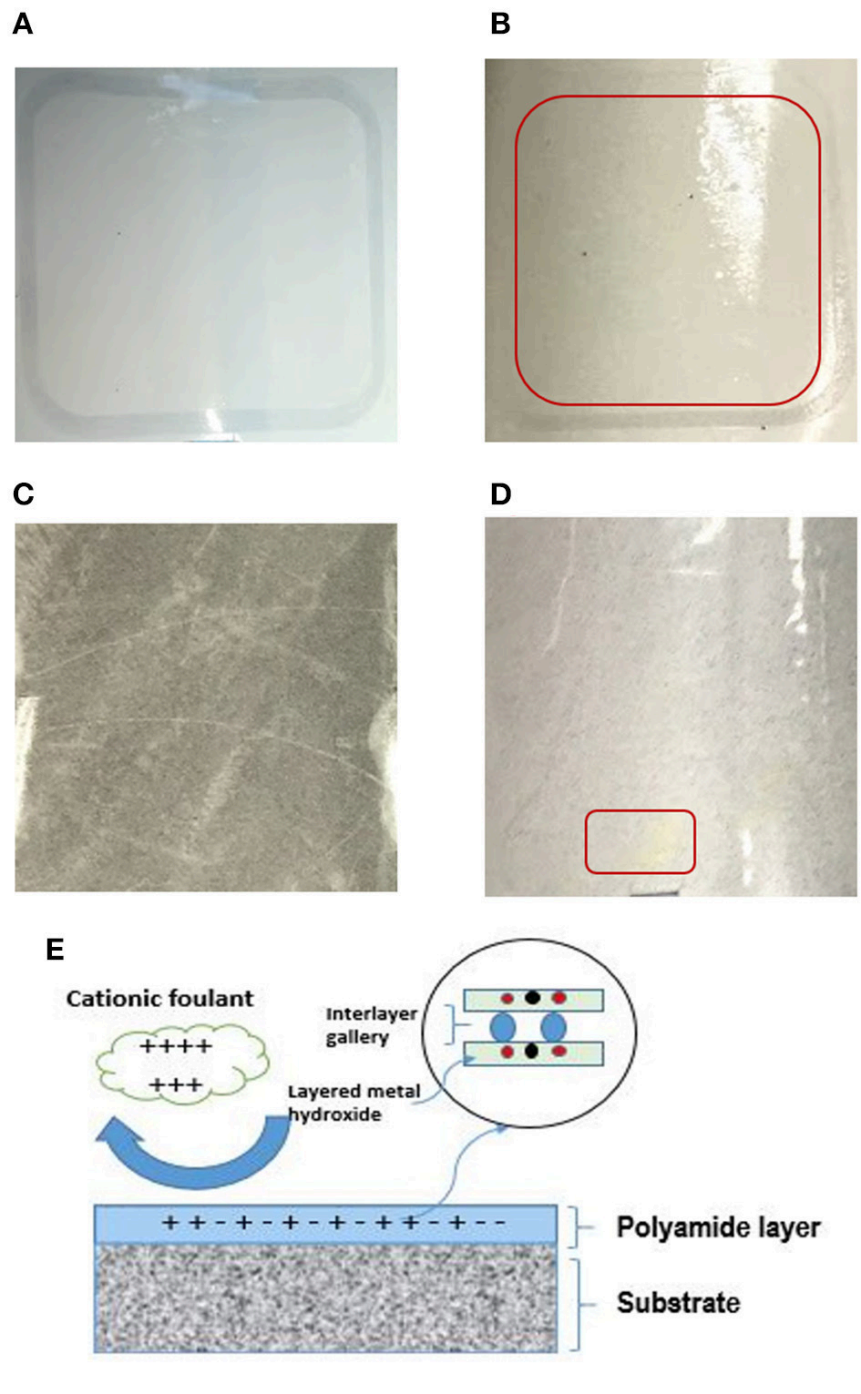
Digital image of membrane before **(A)** TFC and **(C)** TFN 2 and after filtration of BSA **(B)** TFC and **(D)** TFN 2 **(E)** proposed mechanism of anti-fouling for LDH-filled TFN membrane.

Figure 12 displays images of TFC and TFN membranes after 3 h of CTAB filtration. In Figure 12B, it can be seen that the appearance of cloudy stains on top of the TFC membrane indicates possible CTAB attachment. As for the TFN membrane, it can be clearly seen that there is minimal stain as shown by the red square on the surface of the membrane in Figure 12D, as compared to the freshly prepared membrane in Figure 12C. The minimum amount of stain displayed by the TFN membranes could possibly be related to the attraction of CTAB molecules onto the membrane surfaces, while the clear visible stain on the TFC membrane is probably because of the stronger attraction of CTAB due to negative zeta potential. Nevertheless, the TFN membranes show better antifouling resistance than the pristine TFC membranes. The excellent anti-fouling properties of TFN could be ascribed to the increase in hydrophilicity as more water molecules were attracted to membrane surfaces to form a water layer that inhibits the attachment of foulants (Zheng et al., 2017). The results of anti-fouling are also consistent with the lower contact angle measurement of the TFN than the TFC membrane as discussed in section **Characterizations of TFC/TFN Membranes** (Figure 6). Furthermore, the better fouling resistance possessed by TFN membranes could be related to the reduction in membrane surface roughness as previously stated in section **Characterizations of TFC/TFN Membranes** (Figure 5), which helps to mitigate fouling attachment on the membranes.

The proposed mechanism of anti-fouling for LDH-filled TFN membranes is displayed in Figure 12E. As can be seen from the figure, the less negative surface charge of a TFN membrane will repel the cationic foulant (i.e., CTAB) from its surface, thus preventing the deposition of foulants from the active layer. Moreover, since the layered metal hydroxide possess hydroxyl groups in its structure, it helps in facilitating more water molecules on the membrane's surface, thus improving the anti-fouling properties of resultant membranes.

## Conclusions

The successful fabrication of TFN membranes could indicate the potential of nanofillers in water separation processes. The Cu-Al LDH were synthesized based on co-precipitation methods. From the morphological structures, surface charge, and chemical functional group show a successful incorporation of LDH in TFN membranes. The hydrophilicity of membranes was significantly improved as the loading of LDH was increased. The LDH-filled TFN membrane shows an improvement in the separation of inorganic salts under the operating condition of 1,000 ppm solution at 25°C and 7 bar. The optimum loading of 0.1 wt% LDH (TFN 2) recorded a satisfactory water flux of 7.01 L/m^2^h.bar with a higher rejection toward inorganic salts (Na_2_SO_4_, MgSO_4_, MgCl_2_, and NaCl) outperformed the pristine TFC (3.18 L/m^2^h.bar). Furthermore, the LDH-filled TFN membrane exhibits better fouling resistance and water recovery enhancement after CTAB filtration. In conclusion, we believe that the incorporation of LDH nanofillers in a thin film nanocomposite membrane can serve as another alternative to produce high-performance nanofiltration membranes for water separation processes.

## Author Contributions

The experiment in the articles was carried out by MT and IW. MT, NY, WW, and JJ were mainly responsible for writing and editing the articles. AI, FA, KN, and NR were mainly responsible for writing and proofing the articles.

### Conflict of Interest Statement

The authors declare that the research was conducted in the absence of any commercial or financial relationships that could be construed as a potential conflict of interest.
